# Dual roles of endothelial FGF-2–FGFR1–PDGF-BB and perivascular FGF-2–FGFR2–PDGFRβ signaling pathways in tumor vascular remodeling

**DOI:** 10.1038/s41421-017-0002-1

**Published:** 2018-01-16

**Authors:** Kayoko Hosaka, Yunlong Yang, Masaki Nakamura, Patrik Andersson, Xiaojuan Yang, Yin Zhang, Takahiro Seki, Martin Scherzer, Olivier Dubey, Xinsheng Wang, Yihai Cao

**Affiliations:** 1 Department of Microbiology, Tumor and Cell Biology, Karolinska Institute, Stockholm, 171 77 Sweden; 2 Central Research Laboratory, The Affiliated Hospital of Qingdao University, Qingdao, 266071 China; 3 Department of Cardiovascular Sciences, University of Leicester, Glenfield Hospital, Leicester, LE3 9QP UK; 4 NIHR Leicester Cardiovascular Biomedical Research Unit, Glenfield Hospital, Leicester, LE3 9QP UK

## Abstract

Perivascular cells are important cellular components in the tumor microenvironment (TME) and they modulate vascular integrity, remodeling, stability, and functions. Here we show using mice models that FGF-2 is a potent pericyte-stimulating factor in tumors. Mechanistically, FGF-2 binds to FGFR2 to stimulate pericyte proliferation and orchestrates the PDGFRβ signaling for vascular recruitment. FGF-2 sensitizes the PDGFRβ signaling through increasing PDGFRβ levels in pericytes. To ensure activation of PDGFRβ, the FGF-2–FGFR1-siganling induces PDGF-BB and PDGF-DD, two ligands for PDGFRβ, in angiogenic endothelial cells. Thus, FGF-2 directly and indirectly stimulates pericyte proliferation and recruitment by modulating the PDGF–PDGFRβ signaling. Our study identifies a novel mechanism by which the FGF-2 and PDGF-BB collaboratively modulate perivascular cell coverage in tumor vessels, thus providing mechanistic insights of pericyte–endothelial cell interactions in TME and conceptual implications for treatment of cancers and other diseases by targeting the FGF-2–FGFR-pericyte axis.

## Introduction

The tumor microenvironment (TME) is constituted of the extracellular matrix and various cellular components including malignant cells, stromal fibroblasts, inflammatory cells, immune cells, vascular endothelial cells, and perivascular cells^1, 2^. These various cells communicate to each other through cell–cell interactions and production of various growth factors and cytokines^2^. Consequently, TME is probably the richest source of various signaling molecules that often become activated and execute their biological functions on various cell types^3^.

Perivascular cells are often tightly associated with vascular endothelial cells and modulate vascular functions by stabilizing vascular networks, promoting vessel maturation and stability, preventing excessive sprouting, preventing uncontrollable leakage, and modulation of blood perfusion^4–9^. Pericyte coverage on microvessels is regulated by multiple signaling molecules that are produced by endothelial cells and other cell types^10^. Among all known regulatory signaling molecules, the platelet-derived growth factor-BB (PDGF-BB)–platelet-derived growth factor receptor β (PDGFRβ) axis is probably the best-characterized signaling system for perivascular cell recruitment^6, 11^. During the early embryonic development, genetic deletion of *Pdgfb* or *Pdgfrb* in mice produced severe vascular defects of hemorrhages leading to lethality owing to lack of pericytes^6, 11^. In angiogenic vessels, endothelial cells produce PDGF-BB to recruit PDGFRβ^+^ pericytes onto the nascent vasculature. Pericyte recruitment in angiogenic vessels ensures unidirectional sprouting of endothelial cells toward the gradient of angiogenic factors such as vascular endothelial growth factor (VEGF). The PDGF-BB–PDGFR signaling synchronizes with other signaling pathways including the VEGF–VEGF receptor 2 (VEGFR2) and the delta-like 4 (Dll4)–Notch signaling pathways^12, 13^. While the VEGF–VEGFR2 induces vascular sprouting, the Dll4–Notch signaling prevents excessive vascular sprouting in collaboration with the PDGF-BB–PDGFRβ system^14^. Thus, imbalanced expression or activation of each of these signaling components would result in vascular dysfunctions.

Fibroblast growth factor-2 (FGF-2) is a ubiquitously expressed growth factor that displays broad biological functions including angiogenesis through activation of FGF receptors (FGFRs)^15^. There are four subtypes of FGFRs; FGFR1–4 that are all cell-surface tyrosine kinase receptors distributed in various cell types^16^. Despite the long-known functions of FGF-2, its biological functions on perivascular cells, especially in relation to the PDGF-BB–PDGFRβ signaling is unknown. Tumors often produce high levels of FGF-2 to support their growth by stimulating tumor cell proliferation and angiogenesis^9, 16^. In the present work, we show that the FGF-2–FGFR2 signaling augments high-pericyte contents in TME and promotes pericyte coverage in tumor vessels. Mechanistically, FGF-2 triggers both direct and indirect signaling pathways to stimulate pericyte proliferation and recruitment. FGF-2 synchronizes with the PDGF-BB–PDGFRβ signaling pathway by modulating their expression and activation. Thus, targeting the FGF-2 signaling pathway may have profound implications for cancer treatment, drug sensitivity, and possible metastasis.

## Results

### FGF-2 markedly modulates the pericyte content in tumors

**Fig. 1:**
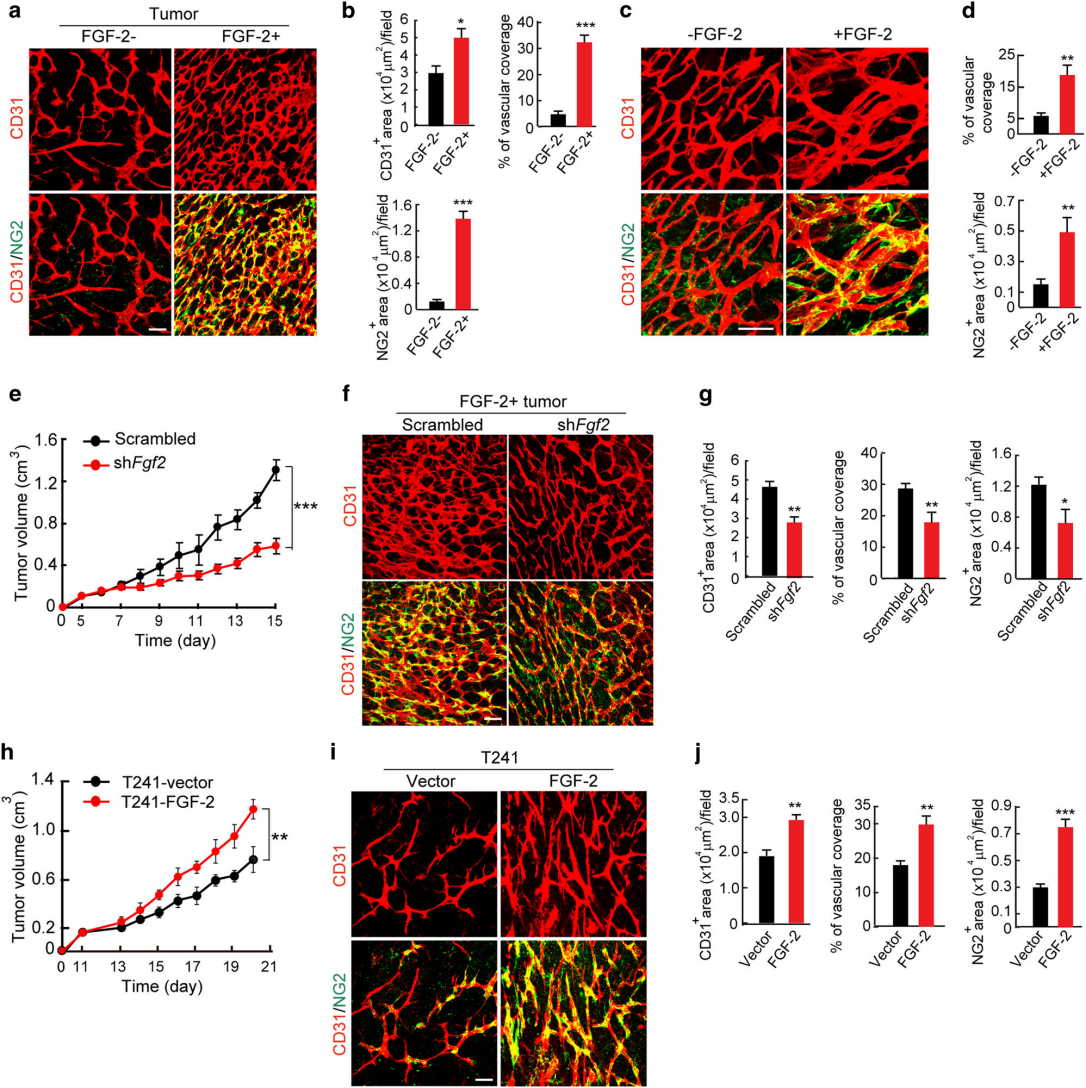
FGF-2-induced angiogenesis, pericyte recruitment, and tumor growth in vivo. **a** Tumor microvessel and pericyte contents. CD31^+^ endothelial cell (red) and NG2^+^ pericyte (green) signals in FGF-2^+^ and FGF-2^−^ tumors. Bar = 50 μm. **b** Quantification of microvessel density, vascular coverage by pericytes, and NG2^+^ pericyte area (*n* = 7 random fields; *n* = 4 mice for each group). **c** CD31^+^ endothelial (red) and NG2^+^ pericyte (green) signals in matrigels containing FGF-2^-^ tumors with and without FGF-2 protein. Bar = 50 μm. **d** Quantification of vascular coverage by pericytes and NG2^+^ pericyte area in matrigels with and without FGF-2 protein (*n* = 7 random fields; *n* = 4 mice for each group). **e** Tumor growth rates of *scrambled-*shRNA and *Fgf2-*shRNA-transfected FGF-2^+^ tumors (*n* = 4–6 animals/group). **f** CD31^+^ endothelial (red) and NG2^+^ pericyte (green) contents in *scrambled-*shRNA and *Fgf2-*shRNA-transfected FGF-2^+^ tumors. Bar = 50 μm. **g** Quantification of microvascular density, vascular coverage by pericytes, and NG2^+^ pericyte area in *scrambled*-shRNA and *Fgf2-*shRNA-transfected FGF-2^+^ tumors (*n* = 7 random fields; *n* = 4 mice for each group). **h** Tumor growth rates of T241-vector and T241-FGF-2 fibrosarcomas (*n* = 6 animals/group). **i** CD31^+^ endothelial (red) and NG2^+^ pericyte (green) contents in T241-vector and -FGF-2 fibrosarcomas. Bar = 50 μm. **j** Quantification of microvessel density, vascular coverage by pericytes, and NG2^+^ pericyte area in T241-vector and T241–FGF-2 fibrosarcomas (*n* = 7 random fields; *n* = 4 mice for each group). Vessels and pericytes were visualized using whole mount staining. All data as means ± S.E.M. *P < 0.05, **P < 0.01 and ***P < 0.001

To study the role of FGF-2 in modulating pericytes in tumor vessels, we selected two cell lines as FGF-2-negative and -positive tumors for in vivo mice tumor models. 3T3 fibroblasts were genetically propagated to become tumorigenic by introducing H-Ras^17^, and used as FGF-2 negative tumor. The H-Ras-driven tumors contained a negligible content of NG2^+^ pericytes (Fig. 1a and b). Notably, expression of a secretory form of the human *Fgf2* gene in these 3T3-originated tumor cells^18^ led to increased NG2^+^ pericyte signals in tumors, which were associated with tumor microvasculatures (Fig. 1a and b). The identity of NG2^+^ pericytes in FGF-2 positive (FGF-2^+^) tumors was further validated with the αSMA known as one of pericyte markers in tumors^10^. αSMA expressions were co-localized with NG2 positive signals (Supplementary Fig. S1). In addition, FGF-2 significantly stimulated tumor angiogenesis (Fig. 1a and b). To validate these findings in genetic tumor models, we took a pharmacological gain-of-function approach in which FGF-2 negative (FGF-2^-^) tumors were grown in a Matrigel containing recombinant FGF-2 protein. Again, FGF-2 protein in Matrigel potently increased the NG2^+^ pericyte content and pericyte coverage in tumor vessels (Fig. 1c and d). Next, we undertook an shRNA loss-of-function approach to block FGF-2 expression. *Fgf2-*shRNA significantly inhibited tumor growth and tumor angiogenesis relative to the control *scrambled*-shRNA-transfected tumors (Fig. 1e–g; Supplementary Fig. S1). Similarly, *Fgf2-*shRNA also markedly inhibited NG2^+^ and αSMA^+^ pericyte contents in FGF-2^+^ tumors (Fig. 1f and g; Supplementary Fig. S2).

To further validate our findings, we used the T241 fibrosarcoma model^19–23^ to genetically express a secretory form of the human *Fgf2* gene. Similar to 3T3 fibroblast-originated tumors, FGF-2 was able to stimulate tumor growth and angiogenesis compared to those of vector-control tumors (Fig. 1h–j). Marked increases of NG2^+^ and αSMA^+^ pericytes were also seen in this tumor model (Fig. 1i and j; Supplementary Fig. S2). Thus, these findings provide evidence of FGF-2 in promoting the tumor NG2^+^ pericyte content and pericyte recruitment in tumor vessels.

### FGF-2-recruited pericytes modulate vascular functions

**Fig. 2:**
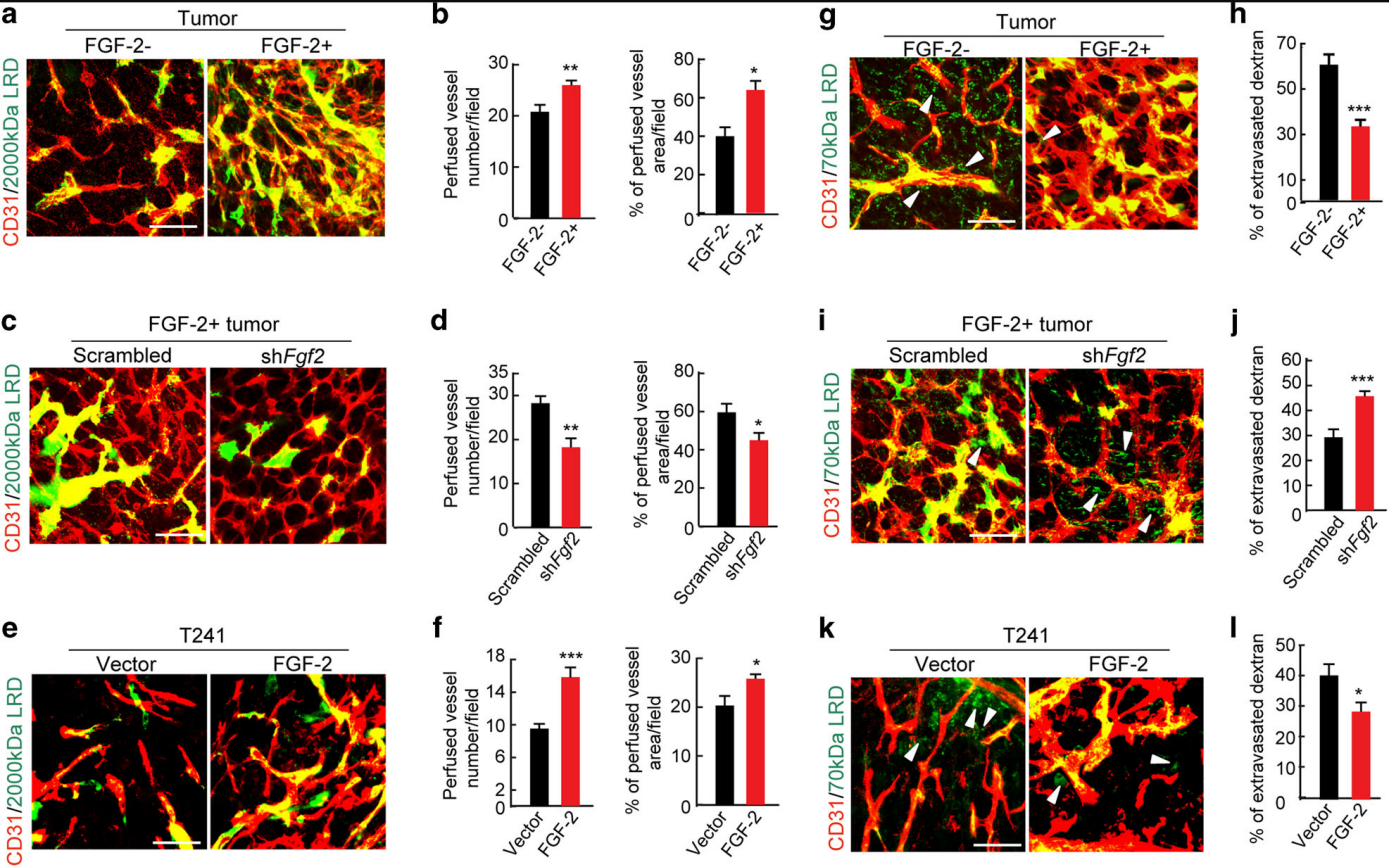
Vascular perfusion and permeability in FGF-2^+^ and FGF-2^-^ tumors in vivo. **a**, **c**, **e** CD31^+^ tumor vasculature (red) and perfusion of 2000-kDa dextran (green) in FGF-2^+^ and FGF^−^, *scrambled-*shRNA and *Fgf2-*shRNA-transfected FGF-2^+^, and T241-vector and T241-FGF-2 tumors. Yellow color indicates double positive signals and perfused vessels. Bar = 50 μm. **b**, **d**, **f** Quantification of blood perfusion in in FGF-2^+^ and FGF^−^, *scrambled-*shRNA and *Fgf2-*shRNA-transfected FGF-2^+^, and T241-vector and T241-FGF-2 tumors (*n* = 10 random fields; *n* = 3 mice for each group). **g**, **i**, **k** CD31^+^ tumor vasculature (red) and leakiness of 70-kDa dextran (green) in FGF-2^+^ and FGF^−^, *scrambled-*shRNA and *Fgf2-*shRNA-transfected FGF-2^+^, and T241-vector and T241-FGF-2 tumors. Arrowheads indicate extravagated dextran (green). Intravascular dextran molecules are in yellow color. Bar = 50 μm. **h**, **j**, **l** Quantification of vascular permeability of 70-kDa dextran in FGF-2^+^ and FGF^−^, *scrambled-*shRNA and *Fgf2-*shRNA-transfected FGF-2^+^, and T241-vector and T241-FGF-2 tumors (*n* = 10 random fields; *n* = 3 mice for each group). Images are shown using whole mount staining. All data as means ± S.E.M.; Student’s *t* test, *P < 0.05, **P < 0.01 and ***P < 0.001

Knowing that FGF-2 potently recruited pericytes onto tumor vessels, we next investigated the functional properties of FGF-2-modulated tumor vessels. Vascular perfusion and leakiness are two major functional parameters to monitor tumor vascular functions^24–27^. In FGF-2^+^ tumors, tumor microvessels were highly perfused with 2000-kD Rhodamine-labeled lysinated dextran (Fig. 2a and b), which were significantly compromised in the *Fgf2*-shRNA-transfected tumors (Fig. 2c and d). These findings were reproduced in the T241 fibrosarcoma tumor model (Fig. 2e and f). Measurement of leakiness of 70-kD Rhodamine-labeled lysinated dextran showed that FGF-2 protected tumor microvasculatures from leakiness (Fig. 2g, h, k and l), consisting with the known functional property of pericytes in prevention of vascular leakage. Again, genetic knockdown of FGF-2 by *Fgf2*- shRNA markedly increased vascular permeability of tumor vessels (Fig. 2i and j). Taken together, these data demonstrate that FGF-2-stimulated pericytes significantly remodel tumor vasculatures and vascular functions.

### FGFR-mediated pericyte recruitment in tumor vessels

**Fig. 3:**
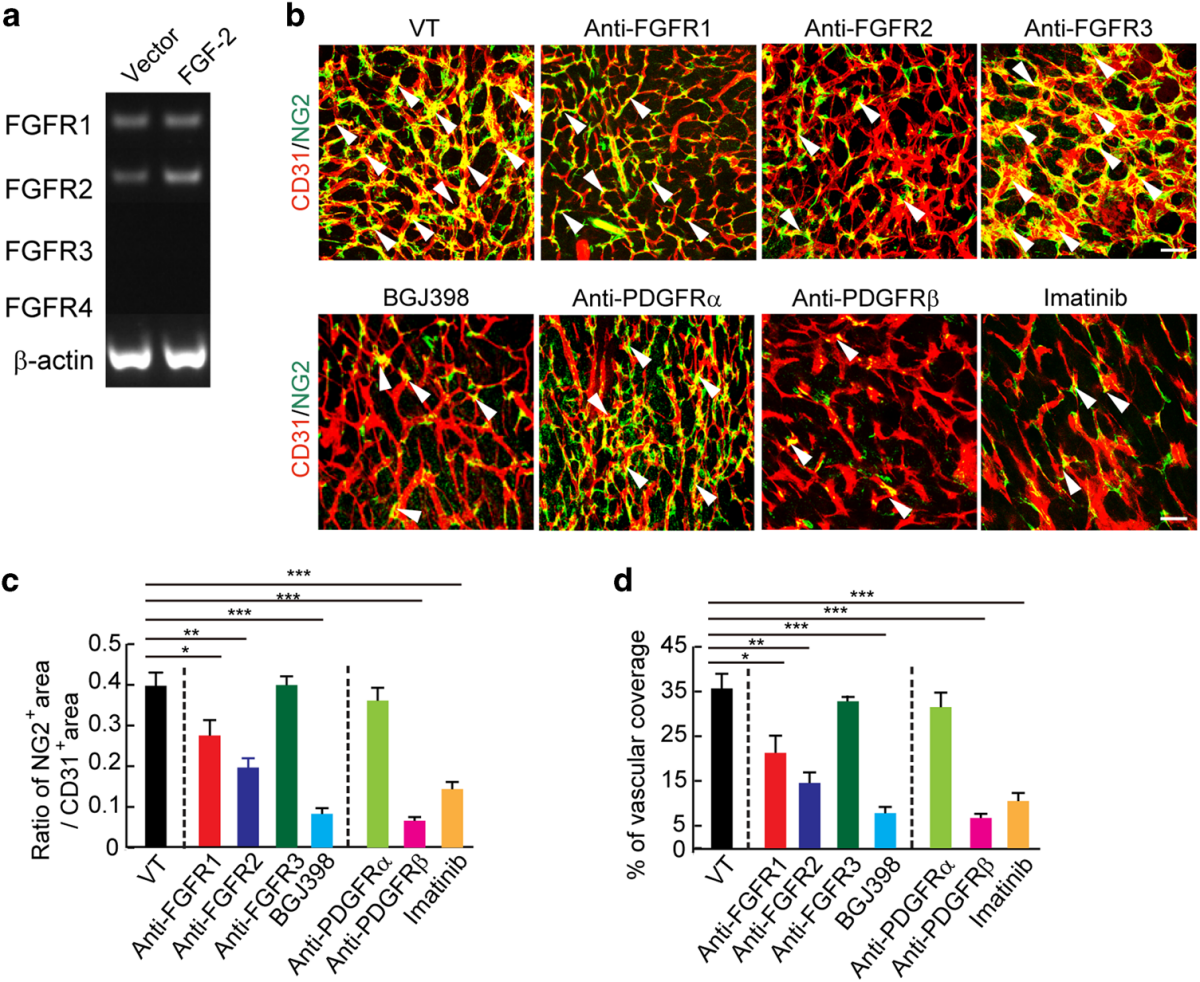
FGFRs and PDGFRs in pericyte recruitment. **a** RT-PCR analysis of mRNA expression levels of FGF receptors in pericytes freshly isolated from T241-vector and T241–FGF-2 tumors using magnetic bead separation. Beta-actin serves as a control. **b** CD31^+^ endothelial (red) and NG2^+^ pericyte (green) signals in vehicle (VT)-, anti-FGFR1 neutralizing antibody-, anti-FGFR2 neutralizing antibody treated-, anti-FGFR3 neutralizing antibody-, BGJ398-, anti-PDGFRα neutralizing antibody-, anti-PDGFRβ neutralizing antibody-, and imatinib-treated FGF-2^+^ tumors. Arrowheads indicate pericyte-associated vessels. Images are presented using whole mount staining. Bar = 50 μm. **c**, **d** Quantification of NG2^+^ pericyte area versus the total CD31^+^ microvessels and vascular coverage. (*n* = 7 random fields; *n* = 4 mice for each group). All data as means ± S.E.M.; Student’s *t* test, *P < 0.05, **P < 0.01 and ***P < 0.001

To define FGF receptors (FGFRs) in mediating FGF-2-induced pericyte recruitment in tumor vasculatures, we isolated fresh pericytes from tumor tissues and immediately checked receptor expression. RT-PCR analysis showed that pericytes in tumors only expressed FGFR1 and FGFR2 receptors and lacked detectable levels of FGFR3 and FGFR4 receptors (Fig. 3a). We next employed a pharmacological approach in vivo to block function of each of the FGFR type using specific anti-FGFR neutralizing antibodies^28^ and a pan-FGFR inhibitor^29–31^. Treatment of FGF-2^+^ tumors with an antibody neutralizing FGFR1 produced only modest but significant inhibition of pericyte recruitment (Fig. 3b–d). An antibody neutralizing FGFR2 significantly reduced the NG2^+^ content of pericytes (Fig. 3b–d), suggesting that FGFR2 is the crucial receptor mediating FGF-2-induced pericyte recruitment in tumors. Consistent with localization studies, blocking FGFR3 with a neutralizing antibody produced virtually no effect on pericyte recruitment (Fig. 3b–d). Strikingly, a pan-FGFR inhibitor, BGJ398^29–31^, nearly completely ablated tumor pericyte contents (Fig. 3b–d). These findings were further validated with an independent set of pericyte marker, αSMA (Supplementary Fig. S3). These data reconcile with the additive effects of FGFR1 and FGFR2 inhibition of pericyte recruitment seen with BGJ398.

As the PDGF–PDGFR system is the best-characterized signaling pathway in pericyte recruitment in angiogenic vessels, we investigated the effects of PDGFRα and  PDGFRβ specific blockades in pericyte recruitment in FGF2^+^ tumor models. Treatment of FGF-2^+^ tumors with a PDGFRα specific neutralizing antibody did not inhibit pericyte contents and vascular coverage (Fig. 3b and d). However, a PDGFRβ specific neutralizing antibody almost completely ablated the pericytes from tumor tissues (Fig. 3b and d, Supplementary Fig. S3), indicating that the PDGFRβ receptor signaling is involved in FGF-2-induced pericyte recruitment. Similarly, imatinib as a tyrosine kinase inhibitor for PDGFRs also markedly inhibited pericytes recruitment in tumors (Fig. 3b and d, Supplementary Fig. S3). These findings show that the PDGFRβ signaling is critically involved in the FGF-2–FGFR1, 2-mediated pericyte recruitment in tumors.

### FGFR2-dependent pericyte proliferation

**Fig. 4:**
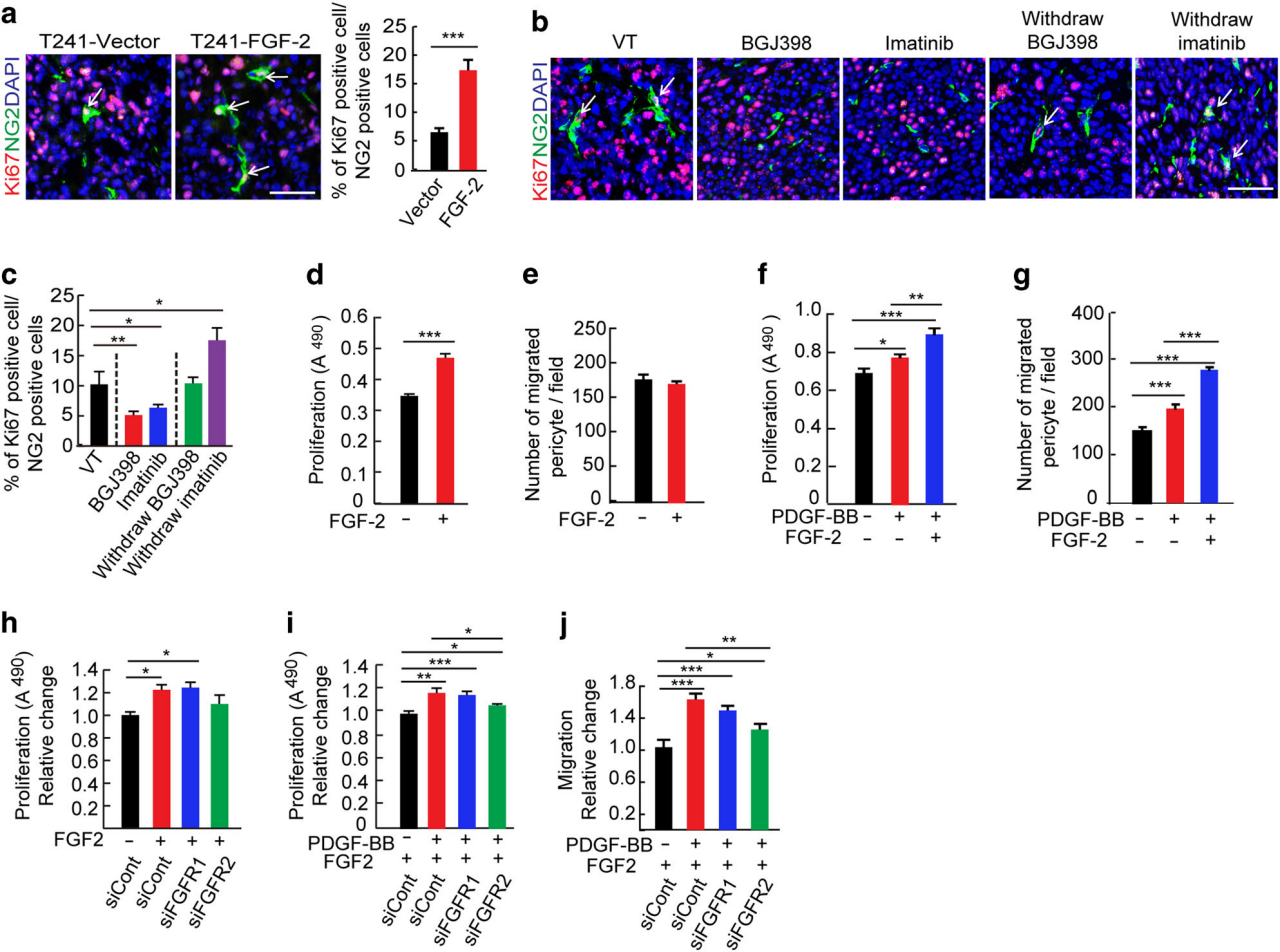
Interplay between the FGF-2–FGFR2 and PDGF-B–PDGFR signaling in perivascular functions. **a** Ki67^+^ (red) and NG2^+^ pericyte (green) in T241-vector and T241–FGF-2 tumor tissue. Cell nuclei were counterstained with DAPI (blue). Allows point to Ki67^+^-NG2^+^ double positive pericytes. Bar = 50 μm. Images are obtained using immunohistochemistry. Quantification of percentages of Ki67^+^-NG2^+^ double positive pericytes tumors (*n* = 15 random fields; *n* = 4 mice for each group). **b** Ki67^+^ (red) and NG2^+^ pericyte (green) in vehicle (VT)-, BGJ398-, and imatinib-treated FGF-2^+^ tumor tissue. Allows point to Ki67^+^-NG2^+^ double positive pericytes. Withdrawal experimental settings were performed at day 6 after drug cessation. Images are acquired using immunohistochemistry. Bar = 50 μm. **c** Quantification of percentages of Ki67 ^+^-NG2^+^ double positive pericytes in VT-, BGJ398-, or imatinib-treated FGF-2^+^ tumors (*n* = 15 random fields; *n* = 4 mice for each group). **d** Pericyte in vitro proliferation after FGF-2 stimulation (*n* = 6 samples/group). **e** Pericyte in vitro migration after FGF-2 stimulation (*n* = 6 samples/group) *P* = 0.43. **f** Pericyte in vitro proliferation after PDGF-BB stimulation with or without FGF-2 pretreatment (*n* = 6 samples/group). **g** Pericyte in vitro migration after PDGF-BB stimulation with or without FGF-2 pretreatment (*n* = 6 samples/group). **h** FGF-2-induced proliferation of *scrambled*-siRNA-, *Fgfr1*-siRNA-, or *Fgfr2*-siRNA-transfected pericytes in vitro (*n* = 6 samples/group). **i** PDGF-BB-induced proliferation of *scrambled*-siRNA-, *Fgfr1*-siRNA-, or *Fgfr2*-siRNA-transfected pericytes with or without FGF-2 pretreatment in vitro (*n* = 6 samples/group). **j** PDGF-BB-induced migration of *scrambled*-siRNA-, *Fgfr1*-siRNA-, or *Fgfr2*-siRNA-transfected pericytes with or without FGF-2 pretreatment in vitro (*n* = 6 samples/group). All data as means ± S.E.M.; Student’s *t* test, *P < 0.05, **P < 0.01 and ***P < 0.001. All in vitro experiments were repeated at least twice

To study the functional impact of FGF-2 on vascular pericytes in TME, we measured the proliferative population of pericytes in the T241 fibrosarcoma model in vivo. The NG2^+^-Ki67^+^ double positive population defined proliferating pericytes in tumors. The NG2^+^-Ki67^+^ cell population was markedly increased in the FGF-2-expressing tumors relative to control tumors (Fig. 4a), suggesting that FGF-2 stimulated pericyte proliferation. Again, a FGFR pan inhibitor effectively blocked pericyte proliferation (Fig. 4b and c). Similarly, imatinib also significantly blocked FGF-2-induced pericyte proliferation in the in vivo tumor models (Fig. 4b and c). To investigate if inhibition of pericyte proliferation by these drugs was reversible, tumors were treated with FGFR and PDGFR inhibitors for one week, followed by withdrawal. Cessation of FGFR and PDGFR inhibitors for only 6 days resulted in full recovery of the NG2^+^ contents (Fig. 4b and c), indicating a reversible process of pericyte proliferation. Withdrawal of imatinib resulted in a rebound effect of pericyte recovery, showing a significantly higher NG2^+^ content than non-treated controls (Fig. 4c).

To further validate these findings, we performed pericyte proliferation and migration assays in vitro. Stimulation of primary NG2^+^ pericytes with FGF-2 exacerbated cell proliferation, but not migration (Fig. 4d and e). By contrast, PDGF-BB was able to stimulate pericyte proliferation and migration (Fig. 4f and g). Intriguingly, FGF-2 significantly potentiated PDGF-BB-induced pericyte proliferation and migration relative to PDGF-BB alone-stimulated cells (Fig. 4f and g). To define the receptor type mediating FGF-2-induced pericyte proliferation, we took a genetic loss-of-function approach by knocking down FGFR1 and FGFR2 using their specific siRNAs. Transfection of pericytes with *Fgfr1-*siRNA and *Fgfr2-*siRNA effectively inhibited mRNA expression levels of their specific receptors (Supplementary Fig. S4). siRNA specifically targeting *Fgfr2*, but not *Fgfr1*, significantly inhibited the FGF-2-induced cell proliferation (Fig. 4h). Similarly, *Fgfr2-*siRNA, but not *Fgfr1-*siRNA, significantly inhibited the FGF-2–PDGF-BB combination-induced pericyte proliferation and migration (Fig. 4i and j). It was surprising that knockdown of FGFR2 blocked FGF-2–PDGF-BB-induced pericyte migration because FGF-2 lacked an overt effect on cell motility.

### FGF-2 protects PDGFRβ from degradation by a recycling mechanism

**Fig. 5:**
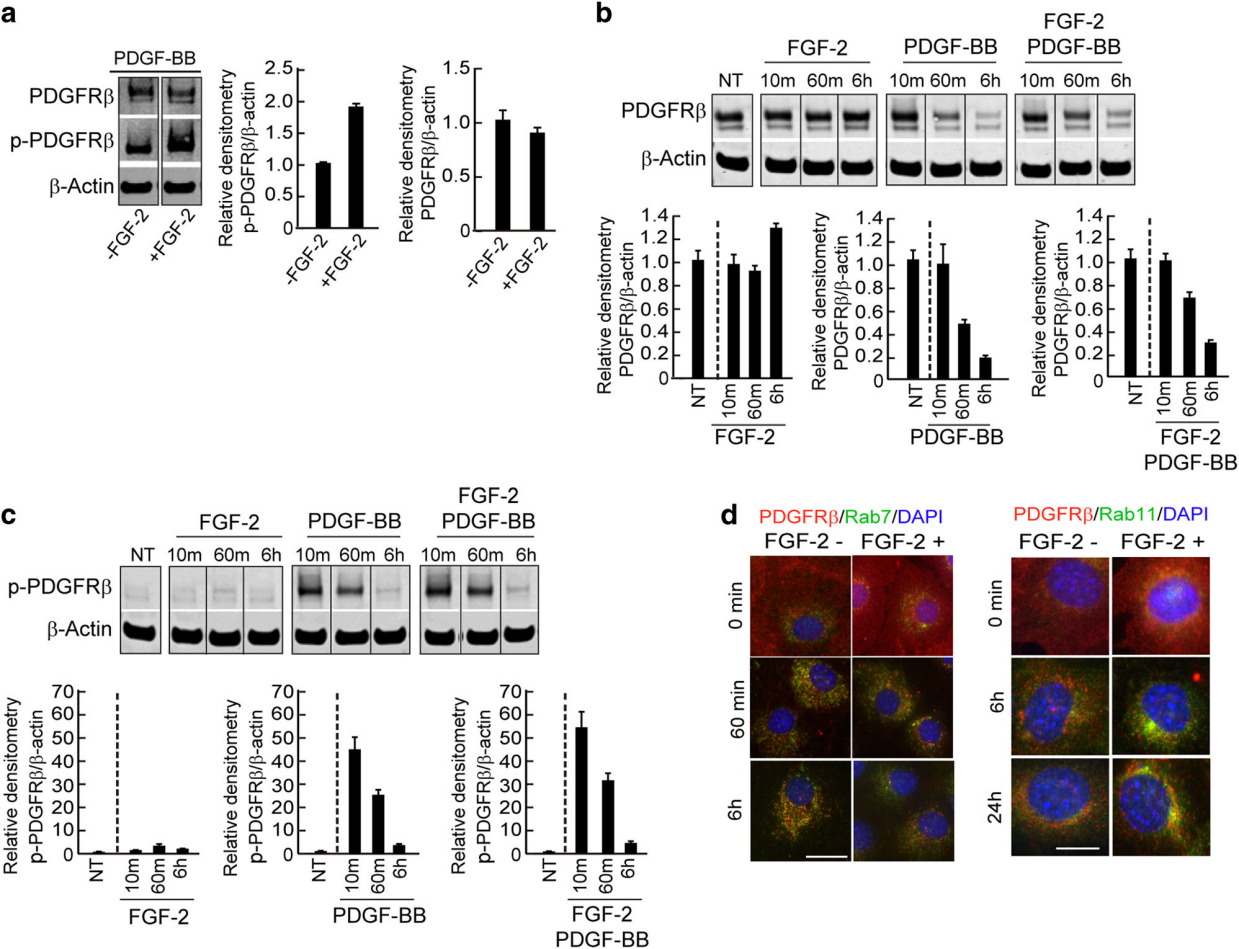
FGF-2-induced sensitization of PDGF-BB–PDGFR signaling by enhancing PDGFRβ recycling. **a** Total and phosphorylated PDGFRβ protein levels in PDGF-BB-stimulated pericytes with or without FGF-2 pretreatment. β-actin levels were used as standard loading controls. Data were presented as means ± S.E.M. (*n* = 3). **b** Time-course analysis of PDGFRβ protein levels in FGF-2-, PDGF-BB-, or FGF-2 plus PDGF-BB-stimulated pericytes. β-actin levels were used as standard loading controls; NT no treatment. **c** Time-course analysis of phosphorylated PDGFRβ protein levels in FGF-2, PDGF-BB-, or FGF-2 plus PDGF-BB-stimulated pericytes. β-actin levels were used as standard loading controls. NT no treatment. **d** PDGF-BB-stimulated and non-stimulated pericytes were stained with specific anti-PDGFRβ, Rab7, and Rab11 antibodies with or without FGF-2 pretreatment. Nuclei were counterstained with DAPI. Bar = 25 μm (left) and 15 μm (right). All data as standardized values. All experiments were repeated three times

We next investigated the mechanisms underlying FGF-2-potentiated biological functions of PDGF-BB on pericytes in vitro. Expectedly, PDGF-BB was able to induce phosphorylation of PDGFRβ in primary NG2^+^ pericytes (Fig. 5a). However, pre-incubation with FGF-2 markedly exacerbated PDGF-BB-induced PDGFRβ phosphorylation in these cells (Fig. 5a). Quantification analysis showed that a nearly two-fold increase of the activated PDGFRβ in FGF-2–PDGF-BB-treated pericytes relative to PDGF-BB-alone-treated cells (Fig. 5a). Thus, FGF-2 enhanced PDGF-BB-induced PDGFRβ activation. However, FGF-2 did not induce mRNA expression levels of PDGFRβ in these cells (Supplementary Fig. S5), suggesting that FGF-2-amplified PDGF-BB–PDGFRβ activation did not occur at the transcription level. Quantification analysis demonstrated an increase of the total PDGFRβ protein in the FGF-2 stimulated pericytes at 6 h (Fig. 5b). In the PDGF-BB-stimulated pericytes, PDGFRβ protein quickly became degraded after only 1 h-stimulation (Fig. 5b). FGF-2 substantially protected PDGFRβ from PDGF-BB-induced degradation (Fig. 5b). Moreover, a large fraction of PDGFRβ remained in its phosphorylated form (Fig. 5c). These findings demonstrate that FGF-2 enhances PDGF-BB-stimulated PDGFRβ activation in pericytes.

To further decipher the molecular mechanism underlying FGF-2-enhanced PDGFRβ activation by PDGF-BB, we studied the PDGFRβ recycling pathway. Rab7 is a late endosome marker and Rab11 is a recycling endosome marker. These two specific markers distinguish endosomal degradation or recycling. In the presence of FGF-2, a marked reduction of Rab7-PDGFRβ double positive signals in PDGF-BB-stimulated pericytes was seen (Fig. 5d). By contrast, the Rab11-PDGFRβ double signals were considerably increased in the FGF-2 plus PDGF-BB-stimulated cells compared to the PDGF-BB alone control (Fig. 5d). These findings indicate that FGF-2 facilitates PDGFRβ recycling to the cell surface and prevents its endosomal–lysosomal degradation.

### FGF-2 augments PDGF-B and PDGF-D production from endothelial cells

**Fig. 6:**
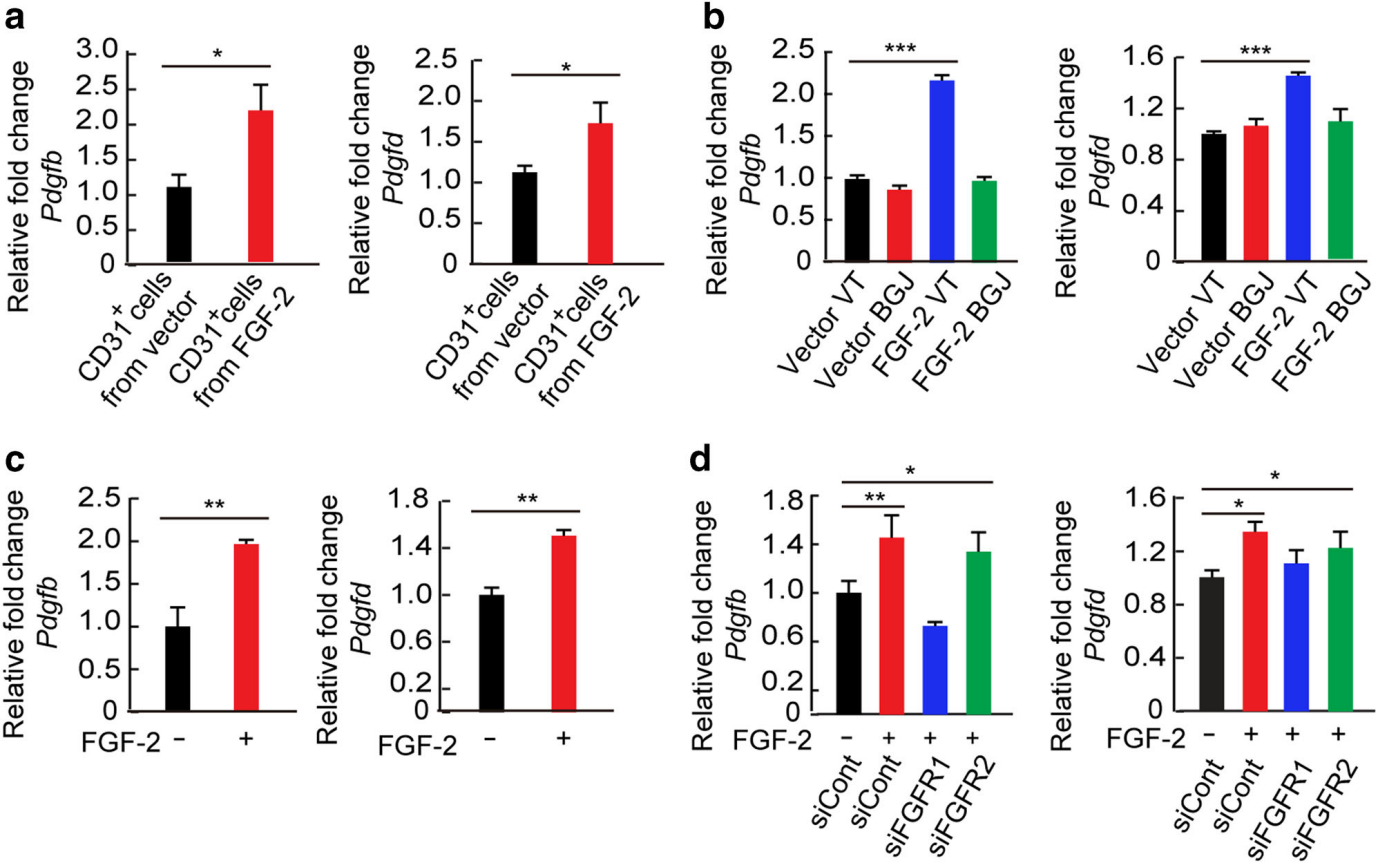
FGF-2-stimulated endothelial *Pdgfb* and *Pdgfd* expression in vivo and in vitro. **a** *Pdgfb* and *Pdgfd* mRNA levels in CD31^+^ ECs freshly isolated from T241-vector or T241–FGF-2 tumors (*n* = 3 samples; *n* = 3 mice for each group). **b** *Pdgfb* and *Pdgfd* mRNA levels in CD31^+^ ECs freshly isolated from VT- or BGJ398-treated T241-vector and -FGF-2 tumors (*n* = 3 samples; *n* = 3 mice for each group). **c** *Pdgfb* and *Pdgfd* mRNA levels in cultivated CD31^+^ ECs in response to FGF-2 stimulation (*n* = 3 samples/group). **d** *Pdgfb* and *Pdgfd* mRNA levels in FGF-2-stimulated cultivated CD31^+^ ECs transfected with *scrambled*-RNA, *siFgfr1*-RNA, or *siFgfr2*-RNA (*n* = 3 samples/group). All data as means ± S.E.M.; Student’s *t* test, *P < 0.05, **P < 0.01 and ***P < 0.001. All in vitro experiments were repeated at least twice

Activation of PDGFRβ requires its binding ligands and PDGF-B and PDGF-D have been identified as PDGFRβ activation binding ligands^32, 33^. It is known that endothelial cells are the rich source for producing these ligands^10^. We analyzed PDGF-B and PDGF-D production in FGF-2-stimulated and non-stimulated endothelial cells. First, we isolated endothelial cells from FGF-2^+^ and control vector tumors and analyzed *Pdgfb* and *Pdgfd* mRNA expression levels. In FGF-2-expressing tumors, *Pdgfb* and *Pdgfd* mRNA expression levels were significantly increased relative to their controls (Fig. 6a). Treatment of FGF-2-expressing tumors with an FGFR-pan inhibitor significantly inhibited *Pdgfb* and *Pdgfd* mRNA expression in endothelial cells (Fig. 6b). To validate these findings, we isolated vascular endothelial cells from non-transfected tumors and treated these cells with FGF-2 in vitro. Again, FGF-2 was able to increase *Pdgfb* and *Pdgfd* mRNA levels, which were inhibited by knocking down of FGFR1 (Fig. 6c and d). The knockdown efficiency was proven to be sufficiently high (Supplementary Fig. S7). These findings show that the FGF-2–FGFR1 signaling is essential for induction of PDGF-B and PDGF-D expression in endothelial cells. We also show that PDGF-DD protein promotes pericyte proliferation and migration (Supplementary Fig. S7).

## Discussion

Multiple angiogenic signaling pathways co-exist in TME and these signaling molecules often communicate each other to collectively determine vascular growth, remodeling, and functions^2^. Thus, angiogenic factors and cytokines do not only vertically bind and activate their specific receptors for signal transduction, they often horizontally modify other signaling pathways that lack direct activation^9, 27, 34, 35^. If such interplay leads to a synergistic effect on vascular functions, co-existence of two angiogenic factors even at low levels would produce greater functional impacts than high levels of single factors. In this study, we provide novel mechanistic insights on functional interplay between the FGF-2–FGFR2 and PDGF-BB–PDGFRβ signaling pathways to recruit perivascular cells in the tumor vasculature.

**Fig. 7:**
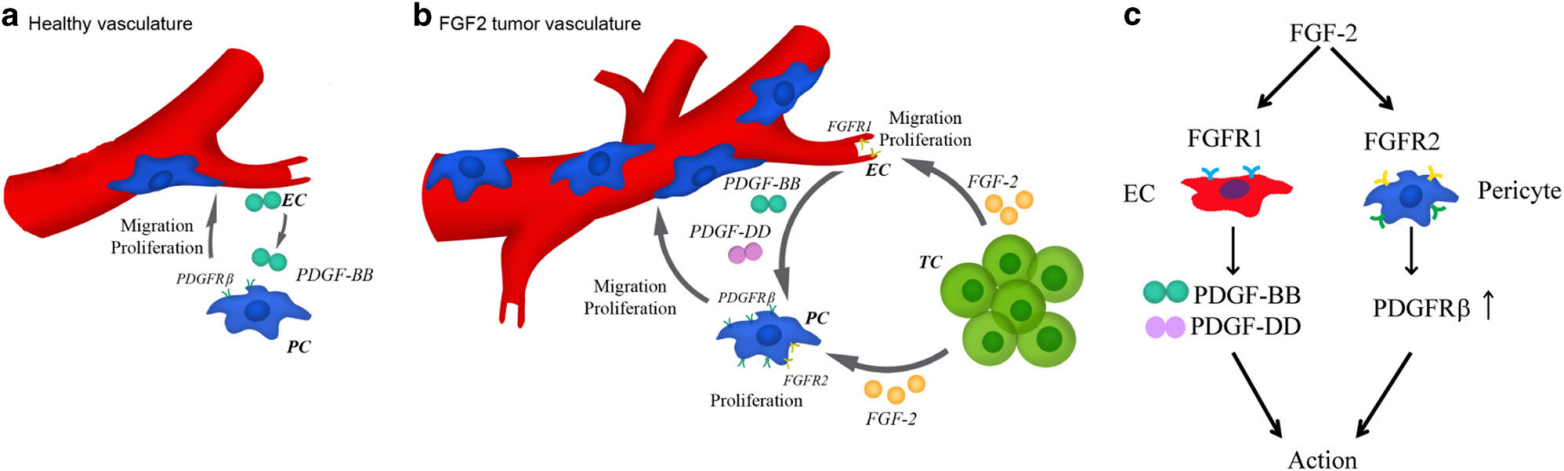
Mechanisms of FGF-2-induced dual effects on endothelial cells and pericytes on vascular remodeling. **a** In healthy vasculatures, angiogenic endothelial cell-derived PDGF-BB promotes pericyte recruitment. **b** In FGF-2^+^ tumors, tumor-derived FGF-2 stimulates endothelial PDGF-BB and PDGF-DD production through the FGFR1 signaling pathway. In addition to endothelial function, FGF-2 stimulates pericyte proliferation and migration through the direct pericyte effect on the FGFR-2 signaling and indirect mechanism by sensitizing PDGFRβ signaling on pericytes. **c** The FGF-2-FGFR1 endothelial and FGF-2-FGFR2 pericyte signaling pathways in vascular remodeling. EC, endothelial cell; PC, pericyte; TC, tumor cell

Although PDGF-BB is a potent proliferative and chemoattractant factor for pericytes and vascular smooth muscle cells, most tumor cells, especially the epithelial cell-derived cancer cells lack detectable PDGF-B expression. Constitutive expression of PDGF-BB can lead to transformation of human cells and combination of rapamycin and imatinib shows a beneficial effect against neoplasia^36–39^. Unlike PDGF-B, FGF-2 is ubiquitously expressed in all tissue cells and often becomes overexpressed in various tumor tissues^15, 16^. In this study, we show that FGF-2 magnifies the PDGF-BB-PDGFRβ signaling at both ligand and receptor levels. At the ligand level, FGF-2 acts on endothelial cells to induce PDGF-B expression (Fig. 7). FGF-2 is a known potent growth factor that stimulates tumor angiogenesis and angiogenic endothelial cells. Previous findings from our laboratory and others showed that FGF-2 and PDGF-BB synergistically induce angiogenesis and promote stable arteriogenesis in ischemic hindlimb models^35, 40^. However, the role of FGF-2- and PDGF-BB-triggered signaling in modulating perivascular cell coverage in angiogenic vessels has not been studied. Another previous study showed that PDGF-BB inhibits FGF-2-induced angiogenesis through a mechanism of heterodimerization between FGF-2 and PDGF-BB^41^. However, it is unclear if such a mechanism also exists in non-endothelial cells such as perivascular cells.

The proliferating population of endothelial cells is especially the rich source of PDGF-B, which recruits pericytes onto angiogenic vessels for maturation. FGF-2 induces endothelial PDGF-B expression through activation of its angiogenic FGFR1 (Fig. 7). In addition, the FGF-2-FGFR1 signaling also induces expression of endothelial PDGF-D, another PDGFRβ-binding ligand. Dual induction of endothelial PDGF-BB and PDGF-DD expression ensures sufficient ligands are available for recruitment of perivascular cells. At the receptor level, FGF-2 utilizes a different receptor, FGFR-2, to ensure the availability of PDGFRβ on pericyte surface for activation (Fig. 7). This mechanism does not involve upregulation of *Pdgfrb* gene expression. The FGF-2-FGFR2 signaling conveys the PDGFRβ protein recycling by preventing its endosomal–lysosomal degradation. In the absence of FGF-2, stimulation of pericytes with PDGF-BB results in rapid internalization and degradation of PDGFRβ. Rapid turnover of PDGFRβ indicates the PDGF-BB-PDGFRβ needs to be switched off in order to control its optimal functions. FGF-2, however, significantly delays PDGFRβ degradation by a mechanism of recycling internalized PDGFRβ back to the cell surface. Reuse of the same receptor is probably a more effective signaling system than protein synthesis of new receptor molecules. The recycling pathway does not need the protein maturation process and the trafficking route is short. Thus, the FGF-2-FGFR2 signaling effectually amplifies PDGF–PDGFRβ signaling in endothelial cells and in perivascular cells. To coordinate with the amplified PDGF–PDGFRβ signaling for recruitment of perivascular cells on to the angiogenic vessels, FGF-2 also directly stimulates pericyte proliferation to increase the pool of recruited cells. By directly and indirectly targeting different steps of perivascular cell proliferation and migration, FGF-2 effectively recruits perivascular cells onto the tumor vasculature. Although our data were obtained from mouse tumor models, these findings are relevant to human tumors because of following reasons: (1) human FGF-2 was used in our animal studies; (2) most human tumors express high levels of FGF-2^42–44^; and (3) FGF-2 is released in the extracellular environment^45, 46^.

What is the functional impact of an increased pericyte content in a tumor? Recruitment of perivascular cells is essential for remodeling and maturation of nascent blood vessels and the mature vasculatures in tumors are better perfused. Indeed, we have found that FGF-2-expressing tumors show improvement of blood perfusion, which would be translated into an accelerated tumor growth rate. In addition, pericytes produce other growth factors and cytokines that either endorse primary tumor growth or promote metastasis. For example, PDGF-BB-stimulated pericytes produce IL-33 that facilitates cancer metastasis by recruiting tumor-associated macrophages^7^. Pericytes also act as stem cells that are able to differentiate into stromal fibroblasts, which promote cancer metastasis^47^. Thus, targeting perivascular cells would provide an important approach for cancer therapy.

Altogether, our findings provide new mechanistic insights of signaling pathways of pericyte recruitment in tumors and drugs targeting these pathways would offer a therapeutic concept for cancer therapy.

## Materials and methods

### Isolation, construction, and maintenance of cells

3T3 fibroblast-derived tumor cells expressing a secreted form of human FGF-2 (named as K1000 and used as FGF-2 positive tumor cells) were obtained and maintained, as previously described^18, 48^. The Ras-transformed 3T3 fibroblasts (named as 3T3ras and used as FGF-2 negative tumor cells) were kindly provided by Dr. Janusz Rak (McGill University, Montreal, Canada)^17^. shRNA-human *Fgf2* gene (*Fgf2*-shRNA) containing plasmids and a lentiviral vector-based expression packaging kit were obtained from GeneCopoeia (Rockville, MD). Transfection was performed according to the manufacturer’s protocol. *Fgf2*-shRNA containing lentiviral particles were produced in 293 T cells by co-transfection with the *Fgf2*-shRNA plasmid and the viral packaging vectors and were harvested from the conditioned medium. *Fgf2*-shRNA containing lentiviral particles were subsequently used to infect K1000 cells, followed by ampicillin selection. The knockdown efficiency was validated using a quantitative PCR method (qPCR). Established cell lines (*Fgfr2*-shRNA k1000) were implanted into mice. A murine fibrosarcoma T241 cell line was used for establishing enhanced green fluorescent protein (eGFP)- and hFGF-2-expressing cell lines. A *Fgf2* cDNA fused to a human a *Il2* secretory leader sequence^18, 48^ was PCR amplified from K1000 cell line and cloned into a construct containing pMXs-internal ribosome entry site-eGFP (pMXs-IG) (a gift from Dr. Toshio Kitamura, The Institute of Medical Science, Tokyo, Japan). T241 cells were transfected with the pMXs-IG-FGF-2-CS23 construct using Lipofectamin2000 according to manufacturer’s instruction. Transfected eGFP-positive FGF-2- or Vector-T241 cells were sorted by flow cytometry (MoFlo XTD; Beckman Coulter). Mouse primary pericytes were isolated from the mouse pulmonary tissue, as previously described^47^. Lung tissues were cut into small pieces and digested 1 h at 37 °C with 0.15% collagenase 2 in PBS (C6885; Sigma-Aldrich). Single cells were incubated on ice for 45 min with 0.1% BSA-PBS, followed by staining with an anti-NG2 rabbit polyclonal antibody (AB5320; Millipore) to bind NG2^+^ pericytes. After washing with PBS, cells were incubated with a Cy3-labeled goat anti-rabbit antibody (AB187; Millipore). Stained cells were sorted by flow cytometry (FACS Vantage/Diva; Becton Dickinson). Mouse endothelial cells were isolated from a T241 tumor. Tumor tissues were cut into small pieces and were incubated for 45 min with 0.15% collagenases 1 and 2 in PBS (C0130, C6885; Sigma-Aldrich). Single cells were stained with a rat anti-mouse CD31 antibody (553370; BD-Pharmingen) on ice for 45 min, followed by incubation for 30 min with a goat anti-rat Alex555 antibody (A21434; Invitrogen). CD31^+^ cells were analyzed and sorted by flow cytometry (MoFlo XTD; Beckman Coulter). Purified cells were cultured for further experiments. All cells are maintained in a Dulbecco’s modified Eagle’s medium (SH30243.01; DMEM, HyClone) supplemented with 10% FBS (SH30160.03; HyClone), 100 U/ml penicillin, and 100 μg/ml streptomycin (SV30010; HyClone) at 37 C with 5% CO_2_. Cells were used within 10 passages after thawing.

### Animal tumor models and drug treatment

C57BL/6 and SCID 6- to 8-wk-old male mice were purchased from the animal facility of the Department of Microbiology, Tumor and Cell Biology at the Karolinska Institutet. All animal studies were approved by the North Stockholm Experimental Animal Ethical Committee. Age- and sex-matched mice were randomly divided to each group. Approximately 1 × 10^6^ tumor cells were subcutaneously injected into the back along the mid dorsal line of each C57BL/6 or SCID mouse. Tumor volume were measured and calculated according to the standard formula (length × width^2^ × 0.52^24, 26, 49–51^. Treatments were started when tumor sizes reached 0.2–0.3 cm^3^ and terminated when control tumors reached the size of 1.0–1.2 cm^3^. A rat anti-mouse FGFR1 neutralizing antibody (40 mg/kg; IMC-A1, ImClone Pharmaceuticals), a rat anti-mouse FGFR2 (IIIc) neutralizing antibody (20 mg/kg; MAB716, R&D Systems Inc.), a rat anti-mouse FGFR3 neutralizing antibody (40 mg/kg; IMC-D11, ImClone Pharmaceuticals), a rat anti-mouse PDGFRα neutralizing antibody (40 mg/kg; IH3, ImClone Pharmaceuticals, kindly provided by Dr. Zhenping Zhu), and a rat anti-mouse PDGFRβ neutralizing antibody (40 mg/kg; 2C5, ImClone Pharmaceuticals, kindly provided by Dr. Zhenping Zhu) were injected intraperitoneally twice per week. BGJ398 (Novartis), a pan-FGFR inhibitor, was dissolved in acetic acid buffer pH 4.6 for 10 min, followed by mixing with PEG300 (50% of final volume) with vortex. BGJ398 was orally given daily to each mouse at a dose of 12.5–30 mg/kg. Imatinib (LC laboratories), a pan-PDGFR inhibitor, was dissolved in PBC and injected intraperitoneally at a dose of 50 mg/kg daily per mouse (*n* = 4–6 animals/group). Vehicle-treated mice served as a control group. In the off-treatment setting, mice were treated with BGJ398 or imatinib for one week and terminated. At day 6 after last treatment, off-treatment groups were sacrificed and tumors were resected for histological examination. A mixture of 1 × 10^6^ 3T3ras tumor cells, 0.5 μg FGF-2 (4037: BioVision), 10 μl heparin (585679; LEO Pharma AB), and 100 μl matrigel were inoculated in the dorsal hump back of each SCID mouse. A mixture of 3T3ras tumor cell–heparin–matrigel was used as a control. At day 6 after inoculation, tumors were removed and fixed in paraformaldehyde (PFA) for histological examination. All animal experiments were terminated by inhalation of a lethal dose of CO2, followed by cervical dislocation.

### Proliferation assay

Primary pericytes (1 × 10^3^) were seeded onto each well of a 96-well plate. Cell proliferation in the absence and presence of 100 ng/ml of FGF-2 (100-18 C; PEPROTECH) (*n* = 5) in DMEM supplemented with 2% FBS was analyzed at 48 h using a MTT (5 mg/ml; M5655; Sigma-Aldrich) method. In some experimental settings, pericytes were stimulated for 48 h with 50 ng/ml of FGF-2 prior to stimulation for 48 h with 10 ng/ml of PDGF-BB (220-BB-050; R&D) (*n* = 6). Densitometry absorbance at 490 nm was measured by a spectrophotometer.

### Chemotaxis assay

Migration of pericytes was measured at 4 h after stimulation (*n* = 6) using the modified Boyden chamber (48-well chambers) method. Pericytes (3 × 10^4^) were added into each of the upper wells and 10 ng/ml of hFGF-2 was placed in the lower wells in DMEM supplemented with 2% FBS. In some experimental settings, pericytes were incubated for 48 h with 50 ng/ml of FGF-2 in a dish prior to stimulation with 10 ng/ml of PDGF-BB for 4 h (*n* = 6). After the boyden chamber was dissembled, migrated cells through 8 μm pores of a polycarbonate membrane (PFB8; Neuro Probe) and attached to the membrane were stained with Giemsa (GS500; Sigma-Aldrich) and photographed by light microscopy with a Nikon DS-QilMC camera (Nikon Corporation, Tokyo, Japan). Migrated cell were captured by microscopy equipped with camera (DS-Fi1; Nikon) and migrated cell number was counted using a software program (NIS-elements D1; Nikon).

### Transient transfection

Pericytes were transfected for 8 h with small interfering RNAs directed against *Fgfr1*(14182; Dharmacon Inc), or *Fgfr2* (14183; Dharmacon Inc). Non-specific *scrambled* small interfering RNAs were used as controls. Transfection was performed according to manufacturer’s protocol (DharmaFECT, Dharmacon Inc.). Transfected cells were analyzed for proliferation and migration using the above-mentioned methods by normalizing with paired-siRNA and paired treatment samples.

### Magnetic bead separation of endothelial cells and pericytes from tumor tissues

Mouse endothelial cells were isolated from vehicle- and BGJ398-treated T241-Vector and T241–FGF-2 tumors to examine PDGF gene expression. Mouse pericytes were isolated from non-treated T241-Vector and -FGF-2 tumors to detect FGFR gene expression. Small pieces of tumor tissues were digested for 45 min at 37 °C with 0.15% collagenases 1 and 2 (Sigma-Aldrich; C0130, C6885). Single cells were stained on ice for 30 min with a rat anti-mouse CD31 antibody (553370; BD-Pharmingen) to bind to endothelial cells or a rabbit anti-mouse NG2 antibody (AB5320; Millipore) to bind to pericytes, followed by incubation for 15 min with a goat anti-rat Cy5 antibody (Millipore; AP183) or a goat anti-rabbit Cy5 antibody (AP132S; Millipore). Cells were washed with PBS and further incubated with anti-Cy5/Alexa Fluor 647 micro beads (130-091-395; Miltenyi Biotec) on ice for 10 min. Magnetic labeled cells were separated using magnetic columns and collected cells were stored in RNAlater (Sigma-Aldrich; R0901) at 4 or −20 °C until further use of gene expression study.

### RNA isolation, RT-PCR, and qPCR

Total RNAs from various cells were extracted using a 2-mercaptoethanol (3148;Sigma-Aldrich)-containing lysis buffer and a RNA extraction kit (K0732; Thermo Scientific). Total RNA concentrations were measured using a nanodrop (Thermo Scientific) and an equal amount of RNA from each sample was applied for cDNA synthesis using a RevertAid cDNA synthesis kit (K1632; Thermo Scientific). RT-PCR was performed for 35 cycles, and each cycle consisted of denaturing at 95 °C for 30 s, annealing at 60 °C for 30 s, and extension at 72 °C for 1 min using an ABI Prism 7500 System (Applied Biosystems). qPCR with SYBR Green (4367659, Life Technologies) was performed using a StepOne-Plus detectable system (Applied Biosystems). qPCR cycles and reactions were performed according to a standard protocol for SYBR Green. All qPCR data were presented as relative quantification and beta actin was used as loading control for RT-PCR analysis. The specific primers used in our experiments were: human *Fgf2* sense: 5′-AGCGACCCTCACATCAAG-3′ and anti-sense: 5′-ATCTTCCATCTTCCTTCATAGC-3′; mouse *Pdgfrb* sense: 5′-TCAACGACTCACCAGTGCTC-3′ and anti-sense: 5′-TTCAGAGGCAGGTAGGTGCT-3′; mouse *Fgfr1* sense: 5′-GGCTACAAGGTACGGTATGC-3′ and anti-sense: 5′-TGGTACGTGTGGTTGATGCTG-3′; mouse *Fgfr2* sense: 5′-GCCCTACCTCAAGGTTATGAAAG-3′ and anti-sense: 5′-GATAGAATTACCCGCCAAGCA-3′; mouse *Fgfr3* sense: 5′-CCCTACGTCACTGTACTCAAGACTG-3′ and anti-sense: 5′-GTGACATTGTGCAAGGACAGAAC-3′; mouse *Fgfr4* sense: 5′-CGACGGTTTCCCCTACGTACA-3′ and anti-sense: 5′-TGCCCGCCAGACAGGTATAC-3′; mouse *Pdgfb* sense: 5′-GGCGAGCGAGTGGGTAGATA-3′ and anti-sense: 5′-TGGAAAGTTGGCTTTGCAGC-3′; mouse *Pdgfd* sense: 5′-CGAGGGACTGTGCAGTAGAAA-3′ and anti-sense: 5′-TTGATGGATGCTCTCTGCGG-3′; and mouse β*actin* sense: 5′-AGGCCCAGAGCAAGAGAGG-3′ and anti-sense: 5′-TACATGGCTGGGGTGTTGAA-3′.

### Whole mount staining

Tumor tissue samples were fixed overnight with 4% PFA and cut into small pieces. Tissues were digested for 5 min with 20 mM proteinase K in 10 mM Tris buffer (pH 7.5), followed by incubation with 100% methanol for 30 min. Tumor tissues were washed with PBS and incubated overnight at 4 °C in 0.3% Triton X-100 PBS containing 3% skim milk. Samples were incubated with a combination of a rat anti-mouse CD31 (1:200; 553370; BD Pharmingen) antibody and a rabbit polyclonal anti-NG2 (1:200; AB5320; Millipore) antibody, followed by incubation with secondary antibodies: an Alexa Fluor 555-labeled goat anti-rat (1:200; A21434; Invitrogen); a Cy5-labeled goat anti-rabbit (1:200; AP187C; Millipore); and Cy5-labeled goat anti-rat (1:200; AP183S; Millipore). After rigorous washing, tissues were mounted using vectashield mounting medium (H1000; Vector Laboratories) and images were taken by confocal microscopy (Nikon C1 Confocal microscope, Nikon Corporation, Japan). Three-dimensional images of tumor vessels were analyzed. Positive signals of CD31 or NG2 area were calculated using an Adobe Photoshop software (CS5; Adobe) program. Vascular coverage was quantified as a percentage of vessels covered by pericytes by calculating overlapping area of CD31 and NG2 double positive signals. Vascular association was counted as the ratio between the total number of pericytes and the number of these with associated vessels.

### Blood perfusion and vascular permeability

Briefly, 1 mg of 2000-kD-lysinated LRD (D7139; Invitrogen) or 1.25 mg of 70-kD-lysinated LRD (D1818; Invitrogen) in 100 ml ddH_2_O was intravenously injected into the tail vein of each mouse. At 5-min or 15-min post-injection, mice were killed by cervical dislocation and tumors were removed. Tumor tissues were fixed overnight with 4% PFA, followed by whole-mount immunostaining. A rat anti-mouse CD31 (1:200; AP183S; Invitrogen,) and a Cy5-labeled goat anti-rat IgG secondary antibody (1:200; AP183S; Invitrogen,) were used for vessel staining. Positive signals were detected by confocal Microscopy (Nikon C1 Confocal microscope; Nikon Corporation, Japan) and Three-dimensional images were analyzed. Vessels perfusion was quantified either by counting vessel number containing 2000-kD-lysinated LRD per field or as a percentage of vessels area comprising 2000-kD-lysinated LRD. The extravasated dextran was calculated as a percentage of 70-kD-lysinated LRD leaked out from vessels by total 70-kD-lysinated LRD per field.

### Immunohistochemistry

Paraffin-embedded tumor issues were sliced into 5-mm thick section and tissue slides were deparaffinized in Tissue-Clear (1466; Sakura) and rehydrated with sequential immerse in 99–95–70% ethanol. Frozen tissue samples embedded in the OCT Embedding Compound (SAKURA) were sectioned using a cryostat, followed by fixation with 4% PFA for 20 min. Tissue slides were stained with a rat anti-mouse endomucin (1:400; 14-5851-85; eBioscience) antibody, a mouse anti-human αSMA (1:200; M0851; clone 1A4; DAKO) antibody, a rabbit anti-mouse NG2 (1:400; AB5320; Millipore) antibody, and a rat anti-mouse Ki67 (1:100; M7248; DAKO) antibody, followed by staining with species-matched secondary antibodies as follows: an Alexa Fluor 555-labeled goat anti-rat (1:400; A21434; Invitrogen), an Alexa Fluor 488-labeled donkey anti-mouse (1:400; A21202; Invitrogen), and an Alexa Fluor 488-labeled donkey anti-rabbit (1:400; A21206, Invitrogen). Positive signals were detected using a fluorescence microscope equipped with a camera (Nikon, DS-QilMC). Images were analyzed using an Adobe Photoshop software (CS5; Adobe) program. Vascular coverage was quantified as a percentage of vessels covered by αSMA^+^ cells by calculating overlapping area of endomucin and αSMA^+^ positive signals.

### In vitro immunostaining

Pericytes grown on glass-slides were treated with 50 ng/ml of FGF-2 in DMEM containing 2% FBS for 48 h prior to stimulation with 10 ng/ml PDGF-BB. Cells were immediately fixed for 20 min with 4% PFA. After washing three times with PBS, cells were incubated for 5 min with PBS containing 0.15% Triton X-100 (Sigma), followed by incubation with 2% skim milk containing PBS for 1 h. Cells were stained overnight with a rat anti-mouse PDGFRβ antibody (1:400; 14-1402-82; eBioscience), a rabbit anti-mouse RAB7 (1:100; 9367; Cell Signaling) antibody, and a mouse anti-human Rab11 antibody (1:100; 05-853; CHEMICON). Positive signals were visualized using an Alexa Fluor 555–conjugated goat anti-rat antibody (1:400; A21434; Invitrogen), an Alexa Fluor 488–conjugated donkey anti-rabbit antibody (1:400; A21206; Invitrogen), and an Alexa Fluor 555–conjugated goat anti-mouse antibody (1:400; A21424; Invitrogen). Tissue samples were mounted using Vectashield containing 4′-6-diamidino-2-phenylindole (H1001; Vector Laboratories) and positive signals were detected using fluorescence microscopy (Nikon).

### Immunoblotting

Pericytes were incubated for 48 h with 50 ng/ml FGF-2 in DMEM containing 2% FBS prior to stimulation with 10 ng/ml PDGF-BB for 10 min. For time-course experiments, cells were treated for 10 min, 1 and 6 h with 50 ng/ml FGF-2, 50 ng/ml PDGF-BB, or a mixture of FGF-2 and PDGF-BB. Non-treated pericytes were used as a control. Soluble proteins from total cell lysates were applied to an SDS-PAGE (NP0301; Invitrogen), followed by wet transferring onto a methanol-activated polyvinylidene fluoride membrane (LC2002, Invitrogen). Membranes were blocked at room temperature with 3% skim milk for 60 min, followed by incubation overnight with a rabbit anti-mouse PDGFRβ (1:1000; 28E1; Cell Signaling) antibody, a rabbit anti-mouse phospho-PDGFRβ antibody (1:1000; G63G6; Cell Signaling), or an anti-mouse β-actin antibody (1:1000; 3700S; Cell Signaling). Membranes were incubated at room temperature for 60 min with a mixture of secondary antibodies consisting of an anti-mouse secondary antibody conjugated with IRDye 680RD (1:15,000; LI-COR; Lincoln) and an anti-rabbit secondary antibody conjugated with IRDye 800CW (1:15,000; LI-COR; Lincoln). Protein signals were captured using an Odyssey CLx system (LI-COR). Full-gel images are shown in Supplementary Fig. S6.

### Statistical analysis

Statistical analysis were performed using the standard two-tailed Student's *t*-test, and *P < 0.05, **P < 0.01 and ***P < 0.001 were considered statistically significant.

## Electronic supplementary material

Supplementary Information

## References

[1] Alderton G (2013). Tumour microenvironment: to me, to you. Nat. Rev. Cancer.

[2] Cao Y, Zhong W, Sun Y (2009). Improvement of antiangiogenic cancer therapy by understanding the mechanisms of angiogenic factor interplay and drug resistance. Semin. Cancer Biol..

[3] Danovi S (2016). Tumour microenvironment: as time goes by. Nat. Rev. Cancer.

[4] von Tell D, Armulik A, Betsholtz C (2006). Pericytes and vascular stability. Exp. Cell Res..

[5] Abramsson A (2002). Analysis of mural cell recruitment to tumor vessels. Circulation.

[6] Lindahl P (1997). Pericyte loss and microaneurysm formation in PDGF-B-deficient mice. Science.

[7] Yang Y (2016). The PDGF-BB-SOX7 axis-modulated IL-33 in pericytes and stromal cells promotes metastasis through tumour-associated macrophages. Nat. Commun..

[8] Cao R (2010). VEGFR1-mediated pericyte ablation links VEGF and PlGF to cancer-associated retinopathy. Proc. Natl Acad. Sci. USA.

[9] Nissen LJ (2007). Angiogenic factors FGF2 and PDGF-BB synergistically promote murine tumor neovascularization and metastasis. J. Clin. Invest..

[10] Abramsson A, Lindblom P, Betsholtz C (2003). Endothelial and nonendothelial sources of PDGF-B regulate pericyte recruitment and influence vascular pattern formation in tumors. J. Clin. Invest..

[11] Hellstrom M (1999). Role of PDGF-B and PDGFR-beta in recruitment of vascular smooth muscle cells and pericytes during embryonic blood vessel formation in the mouse. Development.

[12] Benedito R (2012). Notch-dependent VEGFR3 upregulation allows angiogenesis without VEGF–VEGFR2 signalling. Nature.

[13] Hellstrom M (2007). Dll4 signalling through Notch1 regulates formation of tip cells during angiogenesis. Nature.

[14] Noguera-Troise I (2006). Blockade of Dll4 inhibits tumour growth by promoting non-productive angiogenesis. Nature.

[15] Beenken A, Mohammadi M (2009). The FGF family: biology, pathophysiology and therapy. Nat. Rev. Drug Discov..

[16] Flippot R (2015). FGF/FGFR signalling: implication in oncogenesis and perspectives. Bull. Cancer.

[17] Rak J (1995). Mutant ras oncogenes upregulate VEGF/VPF expression: implications for induction and inhibition of tumor angiogenesis. Cancer Res..

[18] Yayon A, Klagsbrun M (1990). Autocrine transformation by chimeric signal peptide-basic fibroblast growth factor: reversal by suramin. Proc. Natl Acad. Sci. USA.

[19] Hedlund EM (2009). Malignant cell-derived PlGF promotes normalization and remodeling of the tumor vasculature. Proc. Natl Acad. Sci. USA.

[20] Bjorndahl MA (2005). Vascular endothelial growth factor-a promotes peritumoral lymphangiogenesis and lymphatic metastasis. Cancer Res..

[21] Cao R (1999). Interleukin-18 acts as an angiogenesis and tumor suppressor. FASEB J..

[22] Cao R (1999). Suppression of angiogenesis and tumor growth by the inhibitor K1-5 generated by plasmin-mediated proteolysis. Proc. Natl Acad. Sci. USA.

[23] Cao Y (1998). Expression of angiostatin cDNA in a murine fibrosarcoma suppresses primary tumor growth and produces long-term dormancy of metastases. J. Clin. Invest.

[24] Lim S (2016). Co-option of pre-existing vascular beds in adipose tissue controls tumor growth rates and angiogenesis. Oncotarget.

[25] Zhang Y (2016). Endocrine vasculatures are preferable targets of an antitumor ineffective low dose of anti-VEGF therapy. Proc. Natl Acad. Sci. USA.

[26] Iwamoto H (2015). PlGF-induced VEGFR1-dependent vascular remodeling determines opposing antitumor effects and drug resistance to Dll4-Notch inhibitors. Sci. Adv..

[27] Xue Y (2011). PDGF-BB modulates hematopoiesis and tumor angiogenesis by inducing erythropoietin production in stromal cells. Nat. Med..

[28] Ji H (2014). TNFR1 mediates TNF-alpha-induced tumour lymphangiogenesis and metastasis by modulating VEGF-C–VEGFR3 signalling. Nat. Commun..

[29] Guagnano V (2012). FGFR genetic alterations predict for sensitivity to NVP-BGJ398, a selective pan-FGFR inhibitor. Cancer Discov..

[30] Guagnano V (2011). Discovery of 3-(2,6-dichloro-3,5-dimethoxy-phenyl)-1-{6-[4-(4-ethyl-piperazin-1-yl)-phenylamin o]-pyrimidin-4-yl}-1-methyl-urea (NVP-BGJ398), a potent and selective inhibitor of the fibroblast growth factor receptor family of receptor tyrosine kinase. J. Med. Chem..

[31] Wohrle S (2013). Pharmacological inhibition of fibroblast growth factor (FGF) receptor signaling ameliorates FGF23-mediated hypophosphatemic rickets. J. Bone Miner. Res..

[32] Cao Y (2013). Multifarious functions of PDGFs and PDGFRs in tumor growth and metastasis. Trends Mol. Med..

[33] Heldin CH, Lennartsson J (2013). Structural and functional properties of platelet-derived growth factor and stem cell factor receptors. Cold Spring Harb. Perspect. Biol..

[34] Cao Y, Cao R, Hedlund EMR (2008). Regulation of tumor angiogenesis and metastasis by FGF and PDGF signaling pathways. J. Mol. Med..

[35] Cao R (2003). Angiogenic synergism, vascular stability and improvement of hind-limb ischemia by a combination of PDGF-BB and FGF-2. Nat. Med..

[36] Govindarajan B (2005). Malignant transformation of human cells by constitutive expression of platelet-derived growth factor-BB. J. Biol. Chem..

[37] Govindarajan B (2012). Cooperative benefit for the combination of rapamycin and imatinib in tuberous sclerosis complex neoplasia. Vasc. Cell.

[38] Westermark B, Heldin CH (1986). Platelet-derived growth factor as a mediator of normal and neoplastic cell proliferation. Med Oncol. Tumor Pharmacother..

[39] Heldin CH (1985). Platelet-derived growth factor: mechanism of action and relation to oncogenes. J. Cell Sci. Suppl..

[40] Li J (2010). Synergistic effects of FGF-2 and PDGF-BB on angiogenesis and muscle regeneration in rabbit hindlimb ischemia model. Microvasc. Res..

[41] Faraone D (2006). Heterodimerization of FGF-receptor 1 and PDGF-receptor-alpha: a novel mechanism underlying the inhibitory effect of PDGF-BB on FGF-2 in human cells. Blood.

[42] Folkman J (1988). A heparin-binding angiogenic protein--basic fibroblast growth factor--is stored within basement membrane. Am. J. Pathol..

[43] Ingber DE, Folkman J (1989). Mechanochemical switching between growth and differentiation during fibroblast growth factor-stimulated angiogenesis in vitro: role of extracellular matrix. J. Cell Biol..

[44] Shing Y (1984). Heparin affinity: purification of a tumor-derived capillary endothelial cell growth factor. Science.

[45] Hanahan D, Folkman J (1996). Patterns and emerging mechanisms of the angiogenic switch during tumorigenesis. Cell.

[46] Soutter AD (1993). Basic fibroblast growth factor secreted by an animal tumor is detectable in urine. Cancer Res..

[47] Hosaka K (2016). Pericyte-fibroblast transition promotes tumor growth and metastasis. Proc. Natl Acad. Sci. USA.

[48] Cao R (2012). Collaborative interplay between FGF-2 and VEGF-C promotes lymphangiogenesis and metastasis. Proc. Natl Acad. Sci. USA.

[49] Lim S (2014). VEGFR2-mediated vascular dilation as a mechanism of VEGF-induced anemia and bone marrow cell mobilization. Cell Rep..

[50] Yang Y (2013). Anti-VEGF- and anti-VEGF receptor-induced vascular alteration in mouse healthy tissues. Proc. Natl Acad. Sci. USA.

[51] Hedlund EM (2013). Tumor cell-derived placental growth factor sensitizes antiangiogenic and antitumor effects of anti-VEGF drugs. Proc. Natl Acad. Sci. USA.

