# Supplementary material for: Dual roles of endothelial FGF-2–FGFR1–PDGF-BB and perivascular FGF-2–FGFR2–PDGFRβ signaling pathways in tumor vascular remodeling
Source: Cell Discov. 2018 Jan 16;4:3. doi: 10.1038/s41421-017-0002-1 (PMC5798893; doi:10.1038/s41421-017-0002-1)
Supplement: Supplementary file 1 — Supplementary Information [file 41421_2017_2_MOESM1_ESM.pdf]

Dual roles of endothelial FGF-2-FGFR1-PDGF-BB and perivascular FGF-2-FGFR2-PDGFRβ signaling pathways in tumor vascular remodeling

Kayoko Hosaka[1,2], Yunlong Yang[1,2], Masaki Nakamura[1], Patrik Andersson[1], Xiaojuan Yang[1], Yin Zhang[1], Takahiro Seki[1], Martin Scherzer[1], Olivier Dubey[1], Xinsheng Wang[2] and Yihai Cao[1,2,3,] *

[1]Department of Microbiology, Tumor and Cell Biology, Karolinska Institute, 171 77 Stockholm, Sweden.

[2]Central Research Laboratory, The Affiliated Hospital of Qingdao University, Qingdao, 266071, China

[3]Department of Cardiovascular Sciences, University of Leicester and NIHR Leicester Cardiovascular Biomedical Research Unit, Glenfield Hospital, Leicester, LE3 9QP, UK.

*Correspondence, galley proofs and reprint requests should be addressed to: Yihai Cao, M.D., Ph.D., Department of Microbiology, Tumor and Cell Biology, Karolinska Institutet, 171 77 Stockholm, Sweden. Tel: (+46)-8-5248 7596, Fax: (+46)-8-33 13 99, e-mail: yihai.cao@ki.se

Key words: Pericyte, FGF, PDGF, tumor growth, vascular remodeling

Running title: FGF-2 promotes tumor vessel remodeling

The authors declare no potential conflicts of interest.

Supplementary figures and figure legends

Figure S1

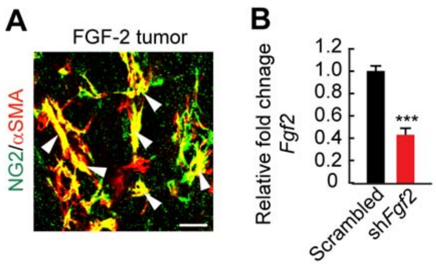

Fig. S1. αSMA and NG2 staining and knockdown efficiency

(A) NG2^{+} (green) and αSMA^{+} (red) staining. Arrowheads indicate double positive signals. Bar = 50 μm. Images are shown using whole mount staining.

(B) *Fgf2* mRNA expression levels in *scrambled-RNA*- and *Fgf2-shRNA*-transfected FGF-2^{+} tumors (n = 3 independent measurements/group).

Data as means ± SEM; Student's *t* test, *** P<0.001.

Figure S2

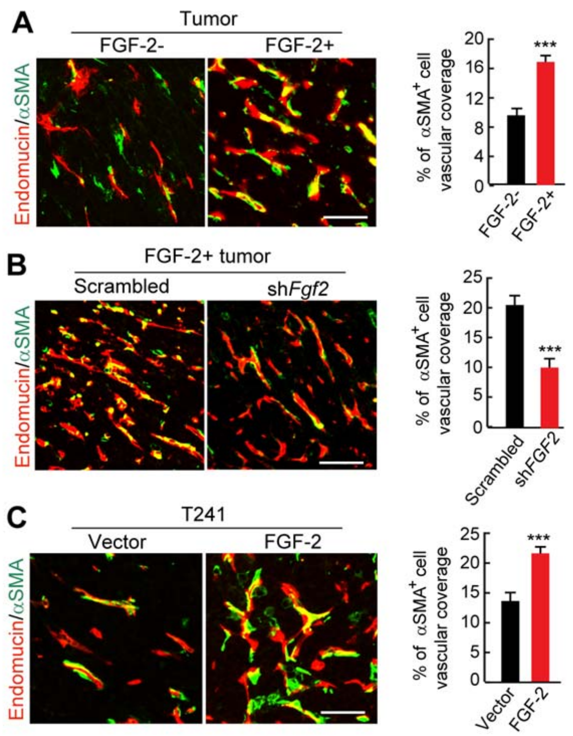

Fig. S2. αSMA and endomucin staining

Endomucin endothelial (red) and αSMA cells (green) in FGF-2$^+$ and FGF$^-$ (A), *scrambled-shRNA* and *Fgf2-shRNA*-transfected FGF-2$^+$ (B), and T241-vector and T241-FGF-2 tumors (C). Bar = 50 μm. Quantification of αSMA$^+$ pericyte coverage (n = 10 random fields/group) are shown respectively. Images are presented using immunohistochemistry method.

Data as means ± SEM; Student's *t* test, *** P<0.001.

Figure S3

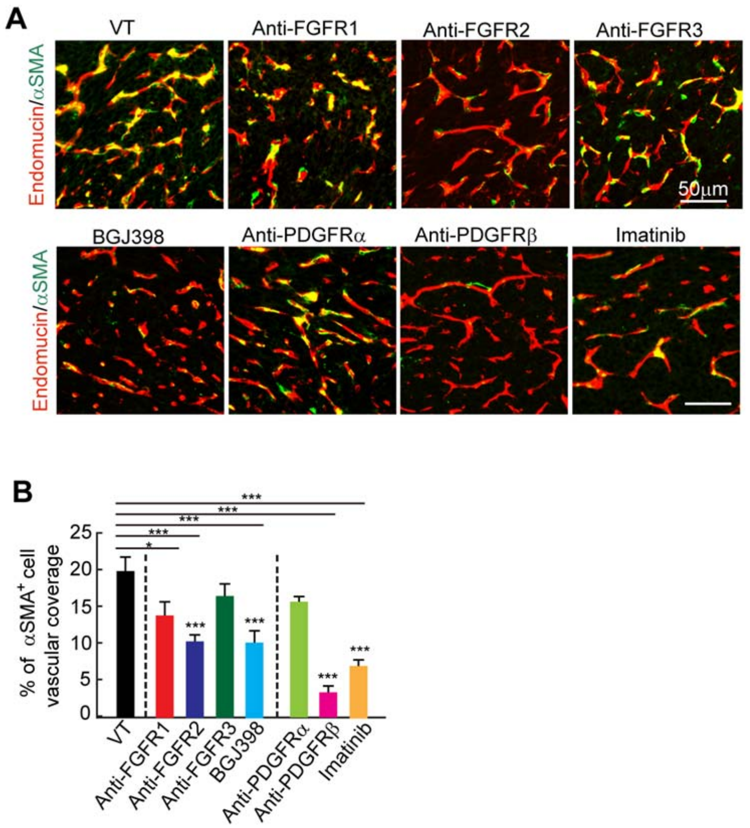

Fig. S3. Effects of FGF and PDGF signaling inhibition in FGF-2 tumors

(A) Endomucin endothelial (red) and αSMA cells (green) in vehicle (VT)-, anti-FGFR1 antibody-, anti-FGFR2 antibody treated-, anti-FGFR3 antibody-, BGJ398-, anti-PDGFRα antibody-, anti-PDGFRβ antibody-, and imatinib-treated FGF-2^+ tumors. Bar = 50 μm. Images are obtained using immunohistochemistry method

(B) Quantification of αSMA^+ pericyte coverage in FGF-2^+ tumor vasculatures (n = 10 fields/group).

All data as means ± S.E.M; Student's *t* test, * P<0.05, ** P<0.01 and *** P<0.001.

Figure S4

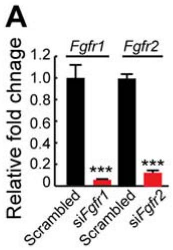

Fig. S4. Knockdown efficiency of *siFGF2-RNA*

(A) *Fgfr1* and *Fgfr2* mRNA expression levels in *Scrambled-siRNA*-, *siFgfr1-RNA*-, and *siFgfr2*-RNA-transfected pericytes (n = 3 independent measurements/group).

Data as means ± SEM; Student's *t* test, *** P<0.001.

Figure S5

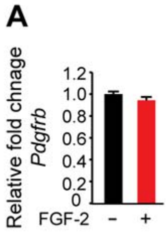

Fig. S5. *Pdgfrb* gene expression level in pericytes.

(A) *Pdgfrb* mRNA expression levels in FGF-2-stimulated pericytes (n = 3 samples /group).

Data as means ± SEM; Student’s *t* test. Experiments were repeated three times.

Figure S6

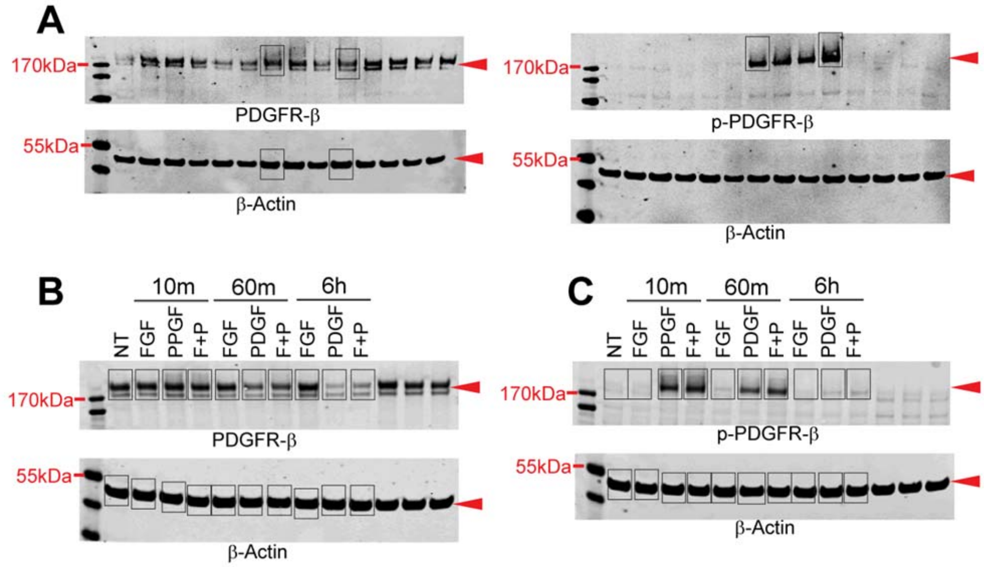

Fig. S6. Original full length western blots used for Fig. 5.

(A) Original western blots used for Fig. 5A.

(B) Original western blots used for Fig. 5B.
NT=no treatment; FGF =FGF-2; PDGF =PDGF-BB; F+P = FGF-2 +PDGF-BB

(C) Original western blots used for Fig. 5C.
NT=no treatment; FGF =FGF-2; PDGF =PDGF-BB; F+P = FGF-2 +PDGF-BB

Cropped parts presented in Fig. 5A-C were marked with black squares.

Figure S7

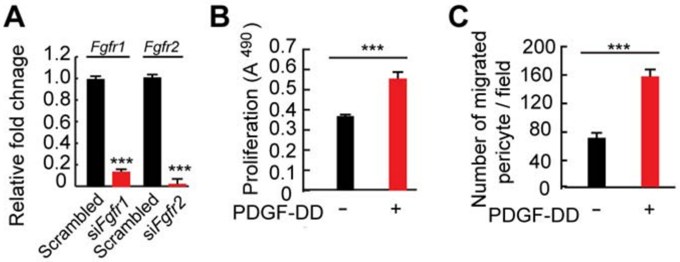

Fig. S7. *Pdgfrb* mRNA expression levels in pericytes.

(A) *Fgfr1* and *Fgfr2* mRNA levels in ECs (n = 3 independent measurements/group).

(B) Pericyte proliferation after PDGF-DD stimulation (n = 6 samples/group).

(C) Pericyte proliferation after PDGF-DD stimulation (n =6 samples/group).

Data as means ± SEM; Student’s *t* test, *** P<0.001. Experiments were repeated three times.
